# Insights into the interconnection of sarcopenia in heart failure

**DOI:** 10.1002/ehf2.15338

**Published:** 2025-06-02

**Authors:** Zhengyang Ge, Yanming Wu, Biao Wang

**Affiliations:** ^1^ Department of Cardiology Suzhou Ninth Hospital Affiliated to Soochow University, Suzhou Ninth People's Hospital Suzhou 215000 China

Sympathetic hyperactivity, an inflammatory system‐triggered immunological response, and catabolism are the hallmarks of heart failure (HF).^1^ A geriatric disorder called sarcopenia is typified by a progressive loss of skeletal muscle mass and function.^2^ The aforementioned underlying causes of HF cause this muscle modification, which is a disease‐specific phenomena that is distinct from ageing‐related change or simple deconditioning.^3^ About 20% of older people with chronic HF have sarcopenia, while over 66% of older patients with acutely decompensated heart failure (ADHF) were shown to be at high risk for the condition.^4^ There appears to be a strong correlation between the pathophysiological processes implicated in HF, age‐related changes in body composition, and sarcopenia, since patients with HF have a considerably greater prevalence of sarcopenia than non‐HF patients.^5^

Through a catabolic shift in muscle homeostasis, increased oxidative processes, increased apoptotic activity, and decreased synthesis of striated muscle growth hormones all contribute to muscle loss in HF patients.^6^ According to previous research, the failing heart affects fat metabolism by secreting atrial natriuretic peptide and brain natriuretic peptide (BNP), which in turn trigger the release of adiponectin and lipolytic effects.^7^ Skeletal muscle atrophy in HF may also be brought on by soluble myostatin secreted from the failing heart.^8^ As demonstrated by Tsuchida *et al*., BNP levels were considerably greater in sarcopenic patients than those without the condition.^9^ Compared to non‐cachectic individuals, patients with HF who experience cachexia are known to exhibit dysregulated neurohormonal signalling, which includes changes in the levels of cortisol, aldosterone, norepinephrine, epinephrine, tumour necrosis factor (TNF), and human growth hormone (GH).^9^ Proteostatic alterations in the muscle of HF patients who are losing weight may be caused by a mismatch between anabolic and catabolic processes, as these changes are generally linked to an increase in catabolism and energy expenditure. A catabolic change in skeletal mass is brought on by immunosenescence dysregulation and low‐grade progressive inflammation, or the so‐called inflammaging. Although there is much information about immune activation in HF patients, many cytokine signals have been more directly connected to muscle atrophy. These include the cytokines interleukin (IL)‐1, IL‐6, and TNF‐α.^4^

Patients with HF often have many comorbidities, such as liver, renal, coronary artery, and cerebrovascular illness.^1^ The development of muscular atrophy linked to metabolic problems is more likely to occur in HF patients.^10^ According to the peripheral hypothesis, although cardiac dysfunction is the main cause of chronic HF, disease progression and symptom severity are influenced by other organs and systems, particularly the immune, endocrine, and renal systems as well as striated skeletal muscle.^11^ Age‐related sarcopenia is the ultimate result of this squandering, which is typified by the atrophy of type II myofibers, decreased capillary density, and fat infiltration.^3^ Muscle wasting results in a reduction in exercise capacity, muscle resistance, and cardiorespiratory fitness. The onset and progression of sarcopenia have also been linked to a number of HF‐related phenomena, such as neurohormonal reactions, nutritional inadequacies, inflammation, inactivity, decreased muscle blood flow, and endothelial dysfunction.^11^ According to earlier research, sarcopenia and its constituents are linked to decreased exercise capacity, sympathoexcitation, and cardiac remodelling, all of which accelerate the development of HF.^12^, ^13^ These imply that sarcopenia and its severity play two roles in relation to HF: They serve as a pathophysiological factor and a severity marker. Therefore, it is thought that sarcopenia and HF are linked and interact and that this vicious cycle has a negative impact on prognosis.

Physical inactivity may be the most obvious connection between sarcopenia and HF.^14^ Reduced functional ability and exercise intolerance are risk factors for sarcopenia development as well as characteristics of HF.^14^ Resistance exercise is still the only treatment approach that has been shown to consistently improve sarcopenia metrics in the senior population.^15^ Sarcopenia is a risk factor for both the reduction in physical ability and the postponement of recovery.^16^ However, the finding by Honda *et al*. did not demonstrate discernible variations in the outcomes connected to in‐hospital rehabilitation and everyday activities.^17^ This is due to the fact that all patients who were hospitalized for HF receive a multidisciplinary team approach that includes early ambulation and exercise training, dietary support, illness education, and pharmaceutical advice.

Additionally, HF patients often suffer from malnutrition to varied degrees, which may be brought on by high inflammatory cytokine levels. As an independent predictor of long‐term mortality, malnutrition is particularly significant in patients admitted for acute HF.^18^ Furthermore, the metabolic consequences would be exacerbated by their increasing decrease in physical activity and related sedentariness. Early nutritional reassessment implementation may have significant benefits for these individuals. Personalized nutritional interventions during and after hospitalization for acute HF may have prognostic benefits, as shown by the Nutritional Intervention Program in Malnourished Patients Admitted for Heart Failure.^19^

In summary, skeletal muscle mass is a significant predictor of outcome for HF patients. Thus, it is critical to identify HF patients with sarcopenia as soon as possible. According to the guidelines of the European Working Group on Sarcopenia in Older People 2, all patients suspected of having sarcopenia should be given the SARC‐F test, a 5‐item self‐report questionnaire that asks patients about their experiences with falls, walking capacity, strength restrictions, getting out of a chair, and stair climbing.^5^ To satisfy the elevated catabolic requirements of the advanced HF patient, optimize physical rehabilitation and nutrition. The widespread use of innovative medication treatments, such as SGLT2i and GLP‐1RA, may cause unanticipated changes in the occurrence of HF sarcopenia.^20^ Prospective studies evaluating this association are desperately needed in order to better understand its mechanism and create tailored treatments.

## Conflict of interest

The authors declare no conflicts of interest.
